# Small Hexokinase 1 Peptide against Toxic SOD1 G93A Mitochondrial Accumulation in ALS Rescues the ATP-Related Respiration

**DOI:** 10.3390/biomedicines9080948

**Published:** 2021-08-03

**Authors:** Andrea Magrì, Pierpaolo Risiglione, Antonella Caccamo, Beatrice Formicola, Marianna Flora Tomasello, Cristina Arrigoni, Stefania Zimbone, Francesca Guarino, Francesca Re, Angela Messina

**Affiliations:** 1 Department of Biological, Geological and Environmental Sciences, University of Catania, Via S. Sofia 64, 95123 Catania, Italy; andrea.magri@unict.it (A.M.); stefania_zimbone@libero.it (S.Z.); 2 we.MitoBiotech S.R.L., C.so Italia 172, 95125 Catania, Italy; fguarin@unict.it; 3 Department of Biomedical and Biotechnological Sciences, University of Catania, Via S. Sofia 64, 95123 Catania, Italy; pierpaolo.risiglione@phd.unict.it; 4 Department of Drug and Health Sciences, University of Catania, Via S. Sofia 64, 95123 Catania, Italy; acaccamo@gmail.com; 5 BioNanoMedicine Center NANOMIB, School of Medicine & Surgery, University of Milano-Bicocca, Via Cadore 48, 20900 Monza, Italy; b.formicola@campus.unimib.it (B.F.); francesca.re1@unimib.it (F.R.); 6 Istituto di Cristallografia, CNR, Via Paolo Gaifami 18, 95126 Catania, Italy; mariannaflora.tomasello@cnr.it; 7 Department of Biology and Biotechnology, University of Pavia, Via Ferrata 9, 27100 Pavia, Italy; cristina.arrigoni01@unipv.it

**Keywords:** amyotrophic lateral sclerosis, SOD1, VDAC1, hexokinase, mitochondria, interfering peptide, high-resolution respirometry

## Abstract

Mutations in Cu/Zn Superoxide Dismutase (SOD1) gene represent one of the most common causes of amyotrophic lateral sclerosis (ALS), a fatal neurodegenerative disorder that specifically affects motor neurons (MNs). The dismutase-active SOD1 G93A mutant is responsible for the formation of toxic aggregates onto the mitochondrial surface, using the Voltage-Dependent Anion Channel 1 (VDAC1) as an anchor point to the organelle. VDAC1 is the master regulator of cellular bioenergetics and by binding to hexokinases (HKs) it controls apoptosis. In ALS, however, SOD1 G93A impairs VDAC1 activity and displaces HK1 from mitochondria, promoting organelle dysfunction, and cell death. Using an ALS cell model, we demonstrate that a small synthetic peptide derived from the HK1 sequence (NHK1) recovers the cell viability in a dose–response manner and the defective mitochondrial respiration profile relative to the ADP phosphorylation. This correlates with an unexpected increase of VDAC1 expression and a reduction of SOD1 mutant accumulation at the mitochondrial level. Overall, our findings provide important new insights into the development of therapeutic molecules to fight ALS and help to better define the link between altered mitochondrial metabolism and MNs death in the disease.

## 1. Introduction

Amyotrophic lateral sclerosis (ALS) is an adult-onset neurodegenerative disorder that affects specifically upper and lower motor neurons (MNs) in brainstem and spinal cord. Symptoms include muscle weakness and atrophy, spasticity and paralysis, and culminate with the death of patients through respiratory failure within 2–5 years from the pathology onset [1].

ALS is predominantly sporadic, although scientific evidence suggests a genetic contribution in all cases, with a Mendelian pattern of inheritance observable in about 10% of patients [2]. Cu/Zn superoxide dismutase (SOD1) was the first gene linked to familial ALS (fALS) [3] and, to date, more than 180 mutations in the 153-codon sequence have been associated with a fifth of the overall inherited cases (ALS on-line database: www.alsod.ac.uk, 2020). Despite this, the exact neurotoxicity mechanism associated to SOD1 mutants is still debated. In the majority of cases, ALS-linked mutations do not cause the loss of dismutase activity, as demonstrated from the extensive studies of SOD1 G93A mutant, but rather the adoption of misfolded conformations and/or a common aberrant hydrophobic behavior, triggering its accumulation [4,5,6,7].

Mitochondrial dysfunction is considered an early and key event for MNs degeneration in ALS. MNs are the key pathological cell-type in ALS: they have axons extending up to a meter long and the maintenance of axonal function is a highly energy-demanding process [8]. It is, therefore, not surprising that correction of the bioenergetic deficit in affected MNs is sufficient to restore axonal length and homeostasis [9].

Alterations in the organelle morphology and/or bioenergetic functions have been observed in tissues from sporadic ALS patients [10,11]. SOD1 mutants impair the activity of complex I of the electron transport chain, calcium uptake and the overall production of ATP [12]. Similar changes have been found in transgenic mice or cell lines expressing SOD1 mutants [13,14,15], where the organelle malfunctioning is accompanied by a re-localization of the predominantly cytosolic SOD1 in the mitochondrial compartment [16,17,18] and, more specifically, onto the cytosolic-facing surface [19]. In this context, the Voltage-Dependent Anion selective Channel 1 (VDAC1), known also as mitochondrial porin, was identified as the main binding site for various SOD1 mutants [20].

VDAC1 is the most abundant protein of the outer mitochondrial membrane (OMM) and plays a crucial role in the regulation of cellular metabolism [21]. VDAC1 is the main member of a family of proteins conserved along the evolution from yeast to human [22,23,24]. Given its β-barrel structure [25,26,27] and its localization at the interface between cytosol and mitochondria [28], VDAC1 modulates the exchanges of ADP/ATP, Krebs’s cycle intermediates and ions (Na^+^, K^−^, Mg^2+^) across the OMM [29,30]. At the same time, VDAC1 is a hub for many cytosolic proteins, such as hexokinases (HK1 and HK2) and Bcl-2 family members, participating in the regulation of apoptosis [31,32,33,34].

The interaction of SOD1 G93A with VDAC1 in ALS has several dramatic consequences for mitochondrial functioning. The addition of SOD1 mutant, but not WT, to reconstituted VDAC1 in artificial membranes blocks the channel conductance [20,35] while, when expressed in transgenic rats, it affects ADP transportation across the OMM [20]. Notably, all these pathological features were exclusively detected in affected tissues but not in liver or brain [17,19,20], suggesting a specific susceptibility of the spinal cord MNs, possibly attributed to the relatively low amount of HKs distinctive of this tissue [20,36]. In this perspective, VDAC1 propensity to interact with SOD1 mutants appears to be increased.

Whereas the N-terminal domain of HK1 mediates the interaction with VDAC1 [37], a small synthetic peptide namely NHK1, correspondent to the first 11 amino acid residues of human HK1, was proposed as an interfering tool for impairing the formation of VDAC1-SOD1 G93A complexes [36]. Cell-free assays indicate that the NHK1 peptide modulates VDAC1 electrophysiological properties by stabilizing the channel in the high conducting state and, if added to the recombinant VDAC1 or purified mitochondria, it significantly interferes with SOD1 G93A binding [36]. In addition, when expressed in an ALS SOD1 G93A cell model by transfection with a plasmid carrying the NHK1 sequence, the peptide partially localized to the mitochondrion and correlated with a significant recovery of the compromised mitochondrial membrane potential [36].

Starting from these evidences, the specific effect of the synthetic NHK1 peptide on mitochondrial functionality was deeply investigated, using the motor neuronal-like cells NSC34 stably expressing SOD1 G93A, a common cell model of ALS. Notably, these cells are characterized by the typical SOD1 mutant accumulation at the mitochondrial level [18], comparable to that seen in transgenic mice or rats expressing SOD1 G93A [19,20], and correlates with a significative impairment of the mitochondrial respiration, as recently observed [38].

In this work, we demonstrate that NHK1 administration recovers the loss of cell viability induced by SOD1 G93A expression in a dose-dependent manner and significantly improves the whole respiratory profile of mitochondria, by specifically increasing the ATP-linked oxygen flows. This is directly related to a decrease in the amount of toxic SOD1 aggregates at the mitochondrial site and a concomitant increase in VDAC1 protein levels. Overall, our findings provide new evidence of the therapeutic value of NHK1 peptide in ALS.

## 2. Materials and Methods

### 2.1. Synthetic NHK1 Peptides

The NHK1 peptide corresponds to the 2–12 amino acid sequence of human HK1 (IAAQLLAYYFT). A non-conjugated and a FITC-labeled peptide, coupled at the C-terminal, were produced by Proteogenix (Schiltigheim, France). NHK1 peptides were stored at −20 °C and dissolved in DMSO.

### 2.2. Cell Cultures, Mantainance, and Viability

The NSC34 motor neuronal-like cell lines were used in their neural-precursor form in continuation with our previous work. Cells stably transfected with pTet-ON plasmid (Clontech, Mountain View, CA, USA) harbouring sequences encoding for human SOD1 WT (NSC34-SOD1WT) or G93A mutant (NSC34-SOD1G93A) were a kind gift of prof. Maria Teresa Carrì (University of Tor Vergata, Rome, Italy) [18]. Cells were cultured in 5% CO_2_ in DMEM/F12 (Sigma-Aldrich, St. Louis, MO, USA) supplemented with 10% tetracycline-free FBS (GIBCO, Waltham, MA, USA), penicillin/streptomycin antibiotic and 200 µg/mL G418 (Carlo Erba, Milan, Italy) for selection maintenance. The maximal expression of SOD1 proteins was achieved by the addition of 2 µg/mL doxycycline (Sigma-Aldrich) to the medium after 48 h. The parental NSC34 cells (CELLutions Biosystem Inc., Duluth, GA, USA) were used as control and cultured according to the manufacturer’s instructions. NSC34-SOD1WT and NSC34-SOD1G93A cells were plated in 96-well plates (10^4^ cells/well) and kept in a controlled environment (37 °C and 5% CO_2_). After 24 h from doxycycline induction, 1, 5, 10, or 50 μg/mL of unlabeled NHK1, previously dissolved in DMSO, were diluted in the culture medium and cells were incubated for additional 24 h. Cell viability was assessed by MTT assay [39]. Parental NSC34 were used as control.

### 2.3. Membrane Permeability Assay by Transwell System

Immortalized human cerebral microvascular endothelial cells (hCMECs) were used as a model of the brain capillary endothelium [40]. hCMEC/D3 cells, provided by Sandrine Bourdoulous of Institut Cochin (Paris, France), were seeded on 12-well Transwell inserts coated with type I collagen (7 × 10^4^ cells/cm^2^) and cultured with 0.5 mL or 1 mL of culture medium in the upper and in the lower chamber, respectively. hCMEC/D3 monolayers integrity was verified by measuring the endothelial permeability of TRITC-dextran and the transendothelial electrical resistance (TEER) with the EVOMX meter, STX2 electrode (World Precision Instruments, Friedberg, Germany). Experiments were performed with a TEER of 40.7 ± 3.7 Ω×cm^2^, together with lower permeability to TRITC-dextran of 6.73 ± 0.91 × 10^−5^ cm/min [41], detected on the seventh day after hCMEC/D3 seeding. The concentration of FITC-NHK1 peptide was 30 μM and was determined in order to exert a neglectable effect on cell viability (i.e., viability loss equal or less than 95%). FITC-NHK1 was added onto the apical compartment and incubated for 3 h. The fluorescence in the basolateral compartment was measured and the endothelial permeability to NHK1 was calculated as previously described [42].

### 2.4. Fluorescence Mycroscopy

NSC34 cells were seeded on 96-wells Cell Carrier Ultra plates (PerkinElmer, Waltham, MA, USA) at a density of 10^4^ cells/well and treated with 10 µg/mL FITC-NHK1 peptide in complete culture medium for 24 h. At the end of the treatments, cells were fixed with 10% formalin and permeabilized with 0.2% Triton X-100 (*v*/*v*) in PBS for 15 min. Following, cells were stained for actin cytoskeleton with Phalloidin Texas Red (1:100 in PBS, 1 h at RT) and nuclei with DAPI (1 µg/mL in PBS, 10 min at RT). All the images were acquired using the Operetta CLS High Content Analysis System (PerkinElmer) equipped with 40X water objective and standard instrument filters. Ten different fields were imaged for each well.

### 2.5. High-Resolution Respirometry

The respiratory capacity of NSC34-SOD1G93A cells was investigated by High-Resolution Respirometry (HRR) in the O2k-FluoRespirometer (Oroboros Instruments, Innsbruck, Austria) with a specific Substrate-Uncoupler-Inhibitor Titration (SUIT) protocol aimed to analyze the different respiratory states and/or the electron transport (ET) system activity [38,43]. Briefly, oxygen consumption in intact cells (ROUTINE) was first analyzed. The dissipative state (LEAK without adenylates) was then determined after cell permeabilization with the mild-detergent digitonin (Sigma Aldrich), used at the final concentration of 4 μM, without compromising mitochondria integrity. The measurement was performed in the presence of 5 mM pyruvate and 2 mM malate (Sigma Aldrich) but not adenylates. The specific contribution of complex I to the OXPHOS was determined with the addition of 10 mM glutamate in presence of a saturating concentration of 2.5 mM ADP (Sigma Aldrich). The following supplementation with 10 mM succinate (Sigma Aldrich) achieved the stimulation of complex II and the measurement of the OXPHOS state. The maximal ET capacity was obtained after titration with 0.5 μM of the uncoupler carbonyl cyanide 3-chlorophenylhydrazone (CCCP, Sigma Aldrich) allowing the proton gradient to complete dissolve. Finally, the residual oxygen consumption (ROX) was accomplished by inhibiting electron transport chain enzymes with the addition of 2 μM rotenone and 2.5 μM antimycin (Sigma Aldrich). All the experiments were performed in mitochondrial respiration buffer Mir05 (Oroboros Instrument) at 37 °C under constant stirring (750 rpm).

### 2.6. Analysis of Respirometric States

Instrumental and chemical background fluxes were calibrated as a function of the oxygen concentration using DatLab software (version 7.4.0.1, Oroboros Instruments). Rate of oxygen consumption corresponding to ROUTINE, LEAK, OXPHOS, and maximal ET capacity was corrected for the ROX and expressed as pmol/s per million cells or as FCRs relative to the maximal ET capacity [44,45,46]. Raw data were reported in Table S1. The ATP-related oxygen fluxes were determined by correcting each specific state for the LEAK respiration and expressed as FCRs. The LEAK-corrected states were also used for the coupling efficiencies calculation, expressing it as a percentage of the capacity in that specific state [44,45].

### 2.7. Cell Lysates and Fractionation

Whole-cell lysates from a near confluent cell population derived from 6-well plates were prepared in a lysis buffer containing 150 mM NaCl, 50 mM Tris-HCL, 1% Triton X-100 pH 7.4, with the addition of protease inhibitor cocktail (Roche, Basel, Switzerland). Enriched mitochondrial and cytosolic fractions were obtained from a near confluent T-75 flask for each condition. 24 h doxycycline-induced NSC34-SOD1G93A cells were treated with 10 μg/mL NHK1 for additional 24 h before fractionation. Approximately 8 × 10^6^ cells were harvested, resuspended in hypotonic fractionation buffer (200 mM mannitol, 70 mM sucrose, 10 mM HEPES, pH 7.5, 1 mM EGTA, pH 8.0) [47] and lysed mechanically in a pre-cooled glass Potter-Elvehjem pestle. Unbroken cells and nuclei were eliminated by centrifugation (700× *g*, 25 min, 4 °C). Supernatants containing the mitochondrial fraction were centrifuged at 7000× *g* for 15 min at 4 °C. Pellets were then lysed in mitochondrial lysis buffer (100 mM Tris-HCl pH 7.4, 1 mM EDTA, 1% Triton X-100, 0.1 mM PMSF) while the supernatant was spun for 30 min at maximum speed at 4 °C to precipitate the majority of the light membrane fraction and obtain a pure cytosolic fraction. Protein concentration was determined by Lowry method.

### 2.8. Western Blot Analysis

Protein samples were separated on NuPAGE Bis-Tris polyacrylamide gels (ThermoFisher, Waltham, MA, USA) and transferred to PVDF membranes (GE Healthcare, Chicago, IL, USA). The membranes were blocked in 5% BSA in PBS with 0.1% Tween-20 and incubated overnight at 4 °C with the following primary antibodies: anti SOD1 (Cell Signaling, Danvers, MA, USA, 1:1000), anti VDAC1 (Abcam, Cambridge, UK, 1:1000), anti HK1 (Cell Signaling, 1:1000), anti β-Tubulin (Cell Signaling, 1:2000), anti COX IV (Cell Signaling, 1:1000), anti SDHA (Abcam, 1:1000), anti β-Actin (Cell Signaling, 1:5000), anti Caspase-3 (Cell Signaling, 1:1000), and anti-cleaved Caspase-3 (Cell Signaling 1:1000). Membranes were incubated with IRDye conjugated secondary antibodies (LI-COR Biosciences, Lincoln, NE, USA, 1:25.000). Signals were detected using Odissey Imaging System (LI-COR Biosciences). Band quantification was performed by densitometric analysis using Image Studio Lite software (version 5.2.5, LI-COR Biosciences).

### 2.9. Real-Time PCR

Total RNA was extracted and purified using Trizol Plus RNA Purification Kit (Life Technologies, Carlsbad, CA, USA) according to manufacturer’s instructions. Residual DNA was removed by DNase I Amplification Grade (Invitrogen, Waltham, MA, USA). Then, RNA was reverse transcribed using High Capacity cDNA Reverse Trascription kit (Applied Biosystem, Waltham, MA, USA) according to manufacturer’ instructions. VDAC1 cDNA concentration was quantitatively analyzed by Real-Time PCR. Three independent experiments were performed in triplicate by using the PowerUp SYBR Green Master Mix (Applied Biosystem). Analysis was performed by using the Mastercycler EP Realplex (Eppendorf, Hamburg, Germany) in 96-well plates. A specific couple of primers for mouse VDAC1 was used (FW: 5′-AAGAAGACCCCGAGACTGGT-3′; REV: 5′-GTTCTCGGAGGCGGTGAC-3′). The housekeeping β-actin gene was used for normalization (FW: 5′-AGCCATGTACGTAGCCATCC-3′; REV: 5′-CTCTCAGCTGTGGTGGTGAA-3′). Quantification of the expression level was performed as previously described [48].

### 2.10. Flow Cytometry Experiments

Mitochondrial mass was evaluated by measuring the fluorescence of MitoTracker Green (ThermoFisher) by flow cytometry. Cells were loaded for 20 min with 200 nM of MitoTracker Green according to the manufacturer instructions. Cells were then collected and analyzed (490/516 nm). A CyFlow ML flow cytometer (Partec, Goerlitz, Germany) system was used. Data obtained were acquired and gated by using the FCS Express software (version 4, DeNovo Software, Pasadena, CA, USA). For each condition examined 20,000 roughly cells were considered.

### 2.11. Cell Transfection

NSC34-SOD1G93A cells were transfected with a modified version of pCMS-EGFP plasmid (Clonthec) carrying the encoding sequence of human VDAC1 or empty vector [28]. Cells were seeded in a 6-well plate and induced with doxycycline. After 24 h, cells were transfected with 2.5 μg DNA per well by Lipofectamine 3000 (Life Technologies) according to manufacturer instructions. VDAC1 expression was verified by Western blot after additional 24 h.

### 2.12. Pull-Down Assay

Recombinant 6xHis-tagged human VDAC1 was expressed, purified and refolded as previously detailed [49,50]. 1 μM of C-terminal His-tagged VDAC1 was immobilized onto Ni^2+^-Sepharose beads (Sigma Aldrich) and incubated with 1 μM NHK1-FITC for 24 h. The unbound peptide was extensively washed and VDAC1 was eluted with 200 mM imidazole. The elution was then loaded onto a Superdex200 (GE Healthcare) and the NHK1-FITC/VDAC1 complex formation was verified by FSEC [51], monitoring elution with a lex/lem = 494/518 nm wavelength, corresponding to the excitation and emission wavelengths of the FITC molecule. For comparison, the VDAC1 elution profile was monitored at 280nm. The 1:1 ratio between VDAC and NHK1 was chosen to avoid that high NHK1-FITC signal in FSEC would mask the signal form the complex.

### 2.13. Docking Simulation

The alpha-helical model of NHK1 has been generated using Modeller (version 9.25, [52]) hereafter called NHK1mod. Docking simulation has been performed with ZDOCK [53] using both the coordinates of NHK1mod and the available crystal structure of human VDAC1 (PDB: 6G6U). ZDOCK run was setup without constraints and to generate 5000 possible poses. All poses were ranked with ZRANK and the complexes having a calculated ΔG < −70 were retained for further analysis.

### 2.14. Statistical Analysis

All data were statistically analyzed by one-way ANOVA or *t*-test. Analyses were performed by using Prism software (version 9, GraphPad Software, San Diego, CA, USA) and expressed as means or median ± standard deviation (SD). At least three independent experiments were performed. The values * *p* < 0.05, ** *p* < 0.01, *** *p* < 0.001 were taken as significant.

## 3. Results

### 3.1. Assessment of Membrane Permeability to NHK1 Peptide

**Figure 1:**
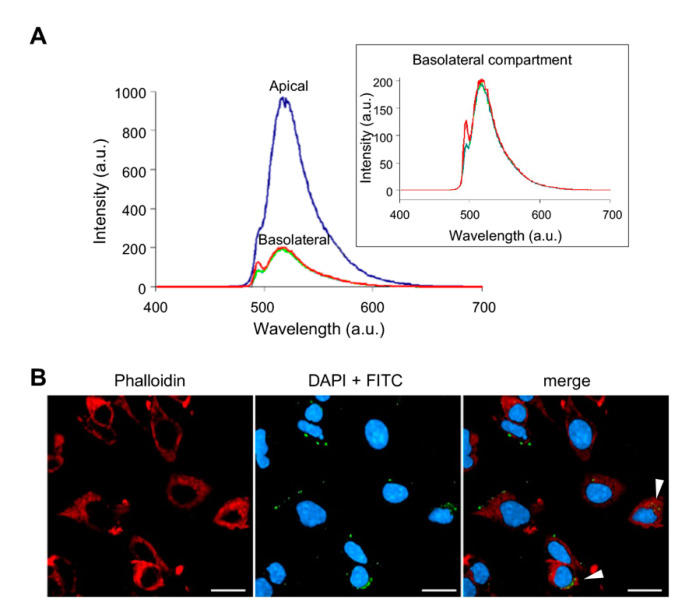
NHK1 peptide crosses biological membranes. (**A**) Analysis of NHK1 peptide permeability in the Transwell system through hCMEC/D3 cells. 30 μM NHK1-FITC was added in the apical compartment and the fluorescence in the basolateral compartment was determined. Colors represent the result obtained for each of three independent experiments. (**B**) Representative images of the NHK1-FITC peptide (in green) uptake by NSC34 cells. In red the Phalloidin Texas Red staining of actin. Arrows indicate the co-localization of signals. Bars represent 20 μm.

NHK1 is a small 11 amino acids length peptide correspondent to the amino terminal residues of the human HK1. According to the GRAVY index [54], NHK1 shows physico-chemical features (i.e., moderate hydrophobicity) that supports its suitability as a cell-penetrating molecules. To evaluate this aspect, the membrane permeability to a fluoresceine isothiocyanate (FITC) labeled peptide (NHK1-FITC) was tested in an in vitro Transwell system integrated with a human brain capillary endothelial cell monolayer (hCMEC/D3), often used as a model of blood–brain barrier. NHK1-FITC was added to the apical compartment and the fluorescence in the basolateral compartment was monitored over time. As shown in Figure 1A, the comparison between spectra obtained from the two compartments indicates that 20.55% of NHK1-FITC has crossed the transwell system, with an endothelial permeability of 5.1 × 10^−4^ cm/min. In parallel, the ability of NHK1 to enter biological membranes was further evaluated in NSC34 cells, following the fluorescence associated to the peptide. As reported in Figure 1B, punctuated FITC signals strictly close to the cell membrane, in the cytosol or in the perinuclear ER/Golgi region were observed. The last is probably an indication of a peptide accumulation site, as seen with other drugs [55]. Furthermore, the target of NHK1, VDAC1, is contained also in the ER membranes [56]. In any case, a partial co-localization with actin was detected (Pearson’s correlation coefficient: 0.417; Manders’ coefficient: 0.265).

### 3.2. NHK1 Ameliorates Cell Viability and Oxygen Consumption in NSC34-SOD1G93A Cells

NSC34 cells stably maintaining a sequence encoding SOD1 G93A represent an inducible model of ALS [18]. The addition of doxycycline induced the expression of the mutant protein and correlated with a loss of cell viability of approximately 25%, observed 48 h after the addition of doxycycline (Figure S1).

**Figure 2:**
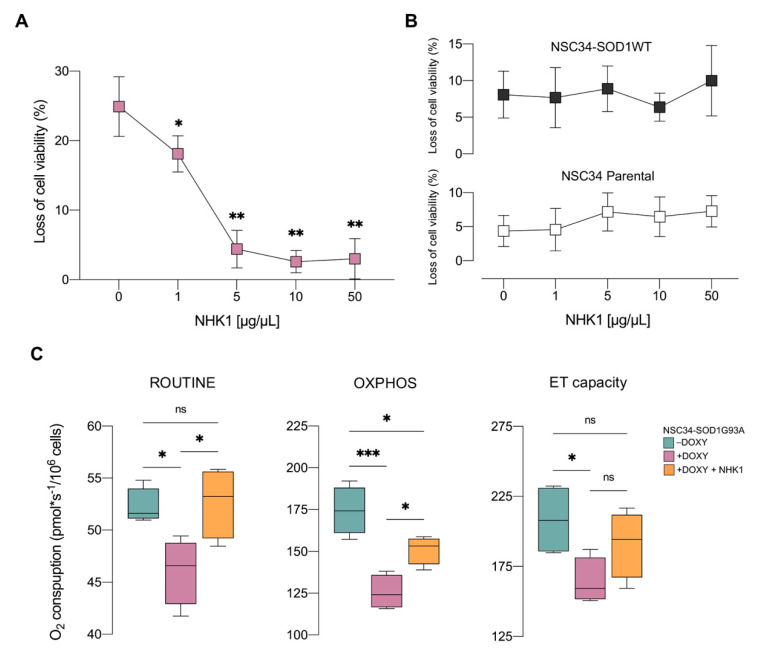
NHK1 counteracts the loss of cell viability and the respiration deficit induced by SOD1 G93A expression. (**A**) Cell viability assay performed in +DOXY NSC34-SOD1G93A in presence of increasing concentration of NHK1 peptide. (**B**) Cell viability control experiments performed in +DOXY NSC34-SOD1WT and in NSC34 parental cells in the presence of increasing concentration of NHK1 peptide. Data are expressed as means ± SD of *n* = 3 independent experiments and analyzed by *t*-test, with * *p* < 0.05 and ** *p* < 0.01 related to untreated NHK1 sample. (**C**) Quantitative analysis of the oxygen consumption of not permeabilized cells (ROUTINE) and of permeabilized cells (OXPHOS and ET capacity). +DOXY NSC34-SOD1G93A cells, previously treated with NHK1 peptide or DMSO, were compared with untreated −DOXY (control). Data are expressed as median or means ± SD of *n* = 4 independent experiments and analyzed by one-way ANOVA, with * *p* < 0.05 and *** *p* < 0.001; ns, not significant.

Given this result, the doxycycline-induced (+DOXY) NSC34-SOD1G93A cells were treated with increasing concentration of unlabeled NHK1 and the effect on the cell viability was monitored. As shown in Figure 2A, the treatment with the synthetic peptide significantly reduced loss of cell viability starting at 1 μg/mL. Remarkably, SOD1 G93A-induced toxicity was completely reversed with 10 μg/mL NHK1. By using the same NHK1 dose, we monitored the activation of caspase-3 as apoptotic marker. However, the cleaved caspase was undetectable and the level of the full-length caspase-3 was comparable among the samples (Figure S2), indicating the absence apoptotic events in our conditions. Furthermore, as control, dose–response curve was repeated in +DOXY NSC34-SOD1WT and in the parental cell line, where no significant variations of the cell viability was observed (Figure 2B). These last experiments indicated the absence of any toxic effect of NHK1 up to the concentration of 50 μg/mL.

The expression of SOD1 G93A is known to affect mitochondrial metabolism in both NSC34 cells and in transgenic mice [16,38,57]. To assess the effect of NHK1 on the organelle functionality, NSC34-SOD1G93A cells were treated with the optimal dose of 10 μg/mL of peptide and oxygen consumption was monitored in different respiratory states by HRR, using a specific substrates-uncoupler-inhibitors titration (SUIT) protocol detailed in Figure S3. First, oxygen consumption was analyzed in intact cells in the presence of endogenous substrates (ROUTINE state). As shown in Figure 2C, the expression of SOD1 G93A resulted in a reduction of oxygen flow of about 12% in comparison to not induced control (−DOXY, *p* = 0.034). This reduction, however, was completely reverted by NHK1 treatment (+14% vs. +DOXY, *p* = 0.024). Then, cells were permeabilized with digitonin, without compromising the mitochondrial membranes integrity, and the oxidative phosphorylation (OXPHOS state) was stimulated by the addition of reducing substrates and ADP. Afterwards, the maximal electron transport (ET) capacity was achieved by uncoupler titration. As shown in Figure 2C, in permeabilized cells SOD1 G93A induced a dramatic reduction of both OXPHOS (−28%, *p* < 0.001) and ET capacity (−21%, *p* = 0.047) in comparison to the -DOXY cells. Again, the treatment with NHK1 peptide ameliorated the oxygen flows of these specific respiratory states and, in particular, the one related to the OXPHOS (+20%, *p* = 0.026 vs. +DOXY).

Overall, these data clearly reveal the ability of NHK1 peptide to counteract both loss of cell viability in a dose-response manner and respiration impairment induced by SOD1 G93A.

### 3.3. NHKI Peptide Improves ATP-Linked OXPHOS Flows but Not Complex I Activity

**Figure 3:**
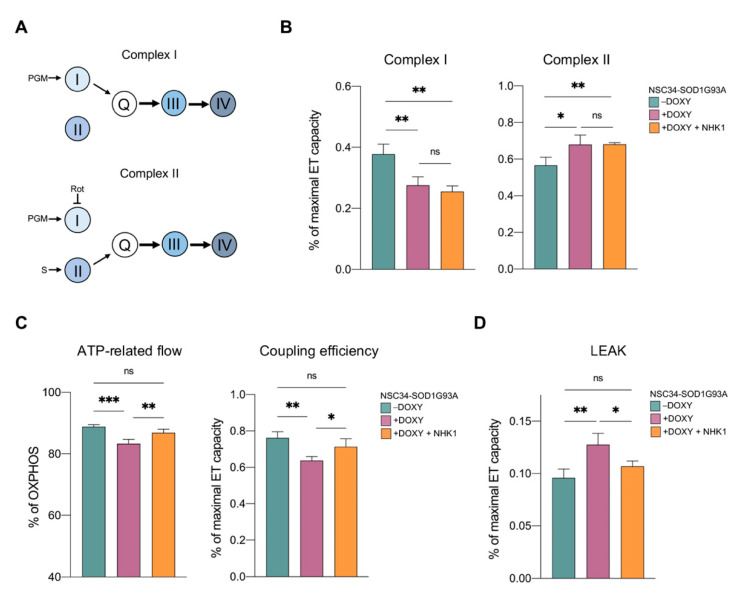
NHK1 peptide improves mitochondrial ATP production but not complex I activity. Oxygen consumption and coupling efficiency of +/−DOXY NSC34-SOD1G93A cells, previously treated with NHK1 peptide or DMSO. (**A**) Schematic representation of the experimental set-up for the analysis of OXPHOS respiration sustained by complex I (upper) or maximal ET capacity sustained by complex II (lower). Pyruvate (P), malate (M), and glutamate (G) are the reducing substrates that stimulate complex I. Succinate (S) stimulates specifically complex II while rotenone (Rot) specifically inhibits complex I. (**B**) Quantitative analysis of complex I and II activity measured as explained in A. (**C**) Quantitative analysis of the OXPHOS flux related to the ADP phosphorylation and the coupling efficiency in the OXPHOS state. (**D**) Quantitative analysis of the LEAK state. Data are showed as FCRs of the maximal ETS capacity or as percentage of the relative state. Data are expressed as means ± SD of *n* = 4 independent experiments and analyzed with one-way ANOVA. * *p* < 0.05, ** *p* < 0.01 and *** *p* < 0.001; ns, not significant.

The expression of SOD1 G93A, but not WT, in NSC34 cells promotes a partial inhibition of complex I, which is accompanied by a compensative increase in the activity of complex II [38]. Notably, this effect is typical of many neurodegenerative disease’s models [43]. To assess whether respiratory chain complexes were a target of NHK1 peptide, activity of complex I and II were investigated by HRR. More than the absolute oxygen consumption, flux control ratios (FCRs) give a better understanding of the contribution of each complex to the maximal ET capacity. As schematized in Figure 3A, electrons flow from complexes I or II to complex III through Q junctions independently of each other. Thus, complex I and II activities can be assayed individually in the presence of specific substrates and/or inhibitors. As shown in Figure 3B, SOD1 G93A expression promoted a significant reduction of oxygen consumption related to complex I (−25%, *p* = 0.005 vs. −DOXY) evaluated in the presence of pyruvate, malate, and glutamate. At the same time, SOD1 mutant correlated with an increase of about 20% in the activity of complex II (*p* = 0.002 vs. −DOXY), assayed after the addition of succinate and rotenone. However, no variation in oxygen flows associated to both complexes was noticed after NHK1 administration in +DOXY samples (Figure 3B), suggesting that the peptide is unable to specifically modulate the activity of complex I and II.

Next, the oxygen flows related to ADP phosphorylation and associated to the OXPHOS respiration were investigated. As reported in Figure 3C, the flux devoted to ATP synthesis was significantly affected by the expression of SOD1 G93A (−16%, *p* = 0.002% vs. −DOXY). Accordingly, the coupling between the electrons transport across respiratory chain complexes and the ADP phosphorylation, the coupling efficiency, was reduced from 88% of the control to 83% of +DOXY cells (*p* < 0.001). In this specific case, the treatment of +DOXY cells with NHK1 ameliorated both the ATP-related OXPHOS flux (+14%, *p* = 0.04 vs. +DOXY) and the coupling efficiency, the latter reaching the value of 87% (*p* = 0.007 vs. +DOXY, Figure 3C).

Finally, by forcing adenylates to leave the cells by mild permeabilization of plasma membranes, the non-phosphorylating respiration (LEAK state) was investigated. LEAK is the dissipative component of the respiration in which the oxygen consumption related to the activity of the respiratory chain compensates for proton leak rather than for ATP production [58]. According with the literature, the presence of SOD1 G93A promoted a LEAK increase as a response or consequence of the mitochondrial dysfunction [38]. Particularly, in our experimental conditions, an increment of about +33% in the LEAK was observed in +DOXY cells (*p* = 0.002 vs. -DOXY, Figure 3D). Remarkably, NHK1 administration completely recovered this dysfunctional parameter up reaching similar level of the control (*p* = 0.016).

HRR data broadly suggest that NHK1 peptide enhances mitochondrial bioenergetic by specifically increasing ATP-related flows and decreasing the dissipative respiration, in an independent manner from complex I or complex II.

### 3.4. NHK1 Peptide Increases VDAC1 Levels While Reduces SOD1 G93A Mitochondrial Accumulation

Being VDAC1 the main gateway of ATP/ADP on the OMM and the target of NHK1 peptide [36], we queried whether the previously observed enhancement in mitochondrial bioenergetics was due to variation in VDAC1 level and/or in its binding partners.

**Figure 4:**
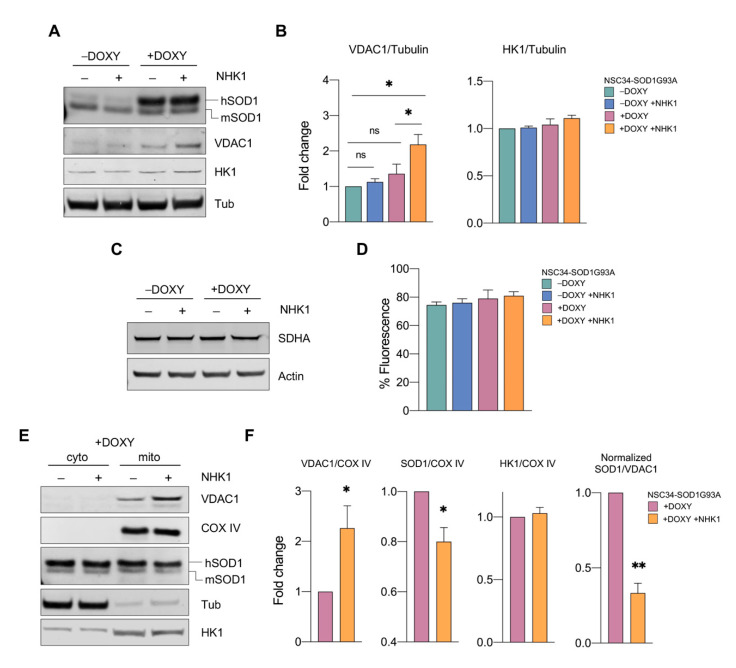
NHK1 promotes VDAC1 overexpression and reduces SOD1 G93A accumulation in mitochondria (**A**) Representative western blot images showing the level of SOD1, VDAC1, and HK1 in total lysates from +/−DOXY NSC34-SOD1G93A treated with NHK1 peptide or DMSO. (**B**) Relative quantification of VDAC1 and HK1 protein levels in the total lysate obtained by densitometry. Tubulin was used as loading control. (**C**) Representative western blot images showing the level of SDHA and actin in total lysates from +/−DOXY NSC34-SOD1G93A treated with NHK1 peptide or DMSO. (**D**) Quantitative analysis of the mitochondrial mass by flow cytometry. The fluorescence of MitoTraker Green was analyzed. (**E**) Representative Western blot images of cytosolic and mitochondrial fractions showing the level of VDAC1, SOD1, and HK1 in +DOXY NSC34-SOD1G93A treated with NHK1 peptide or DMSO. The purity of the fractions was tested by tubulin and COX IV. (**F**) Relative quantification of VDAC1, SOD1, and HK1 in the mitochondrial fraction by densitometry, using COX IV as loading control for the mitochondrial fraction, and the quantification of SOD1 G93A/VDAC1 normalized ratio. All the data in histograms are expressed as means ± SD of *n* = 3 independent experiments and analyzed by *t*-test. Values of * *p* < 0.05 and ** *p* < 0.01 are related to controls; ns, not significant.

The addition of doxycycline to the cells induced SOD1 mutant expression as demonstrated by the detection of a higher band (hSOD1) in addition to the endogenous one (mSOD1) (Figure 4A). The further addition of NHK1 did not change the total SOD1 amount. Conversely, it correlated with an unexpected increase in VDAC1 protein levels of about twice compared to −DOXY cells (Figure 4B). On the contrary, no variations in VDAC1 levels were observed in −DOXY after treatment with NHK1: as shown in Figure 4A, VDAC1 bands were hardly detectable by Western blot and similar signals were observed in total lysate from both cells.

To assess whether VDAC1 increase was a consequence of an improved mitochondrial biogenesis, mitochondrial mass was estimated by analyzing the level of the mitochondrial marker succinate dehydrogenase subunit A (SDHA). Western blot in Figure 4C indicate that no variation in the SDHA levels was detected between samples. To confirm this data, cells were treated with a MitoTracker Green probe, whose uptake within the organelle is independent from the mitochondrial membrane potential [59]. The uptake of the probe was verified by fluorescence microscopy (Figure S4) and quantified by flow cytometry. As reported in Figure 4D, our analysis revealed no significative variations between samples. Altogether, these data suggest that the VDAC1 increase was not correlated with an increase in mitochondrial mass but specifically due to the NHK1 treatment. Notably, a similar increment was detected also for VDAC1 mRNA, as revealed by real-time PCR (Figure S5), suggesting that NHK1 treatment induced somehow the overexpression of VDAC1 gene. Finally, we investigated the levels of endogenous HK1. As reported, NHK1 did not affect HK1 amount in any conditions tested (Figure 4A,B).

As previously demonstrated, the addition of NHK1 peptide to the recombinant VDAC1 or purified mitochondria prevents the binding between SOD1 G93A and VDAC1 [36]. To evaluate if this was the case also in the NSC34-SOD1G93A, we measured the level of proteins of our interest in the mitochondria. As expected, VDAC1 was detected only in the mitochondrial fraction (Figure 4E). By using COX IV as loading marker, we observed an increment of about 2 times of VDAC1 in NHK1 treated cells compared to untreated control (Figure 4F). SOD1 G93A was instead distributed between the mitochondrial and the cytosolic fractions (Figure 4E). Interestingly, the treatment with NHK1 peptide significantly reduced the level of SOD1 mutant in mitochondria of about 20%, possibly as a result of its direct interaction with VDAC1 (Figure 4F). Again, no differences in HK1 were observed at the mitochondrial level.

The simultaneous increase of VDAC1 together with the decrease of SOD1 mutant in the mitochondrial fraction corresponds to a ~67% reduction of SOD1 G93A/VDAC1 ratio (Figure 4F), suggesting that NHK1 peptide affects significantly the formation of toxic aggregates on the cytosolic surface of mitochondria.

### 3.5. VDAC1 Overexpression Is Not Sufficient to Counteract SOD1 G93A Toxicity

**Figure 5:**
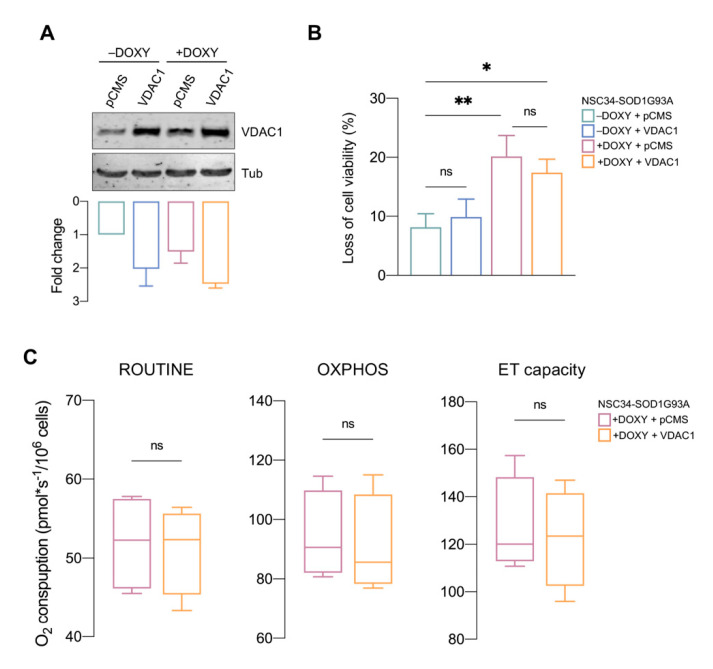
VDAC1 overexpression does not support the mitochondrial bioenergetic of NSC34-SOD1G93A cells. (**A**) Representative Western blot images of total lysates from +/−DOXY NSC34-SOD1G93A transfected with empty plasmid pCMS or plasmid carrying VDAC1, and the relative quantification. Tubulin was used as loading control. (**B**) Cell viability assay of +/−DOXY NSC34-SOD1G93A transfected as in A. Data are expressed as median ± SD of *n* = 3 independent experiments and analyzed by one-way ANOVA. Values of * *p* < 0.05 and ** *p* < 0.01 were taken as significant. (**C**) Quantitative analysis of the oxygen consumption in intact (ROUTINE) or permeabilized cells (OXPHOS and ET capacity) of +DOXY NSC34-SOD1G93A cells transfected with empty plasmid pCMS or plasmid carrying VDAC1. Data are expressed as median ± SD of *n* = 4 independent experiments and analyzed by *t*-test; ns, not significant.

According to the previous results, we questioned whether this unexpected increment in the VDAC1 levels was the principal responsible for the recovery of cell viability and respiration profile observed in the presence of NHK1 peptide. In this perspective, NSC34-SOD1G93A cells were transiently transfected with a plasmid carrying the encoding sequence of VDAC1 and producing a mitochondrially targeted protein [28]. Transfection was performed in order to obtain an increment of VDAC1 level of about 2 times, mimicking the exact condition attained with the NHK1 treatment (Figure 5A). In this condition, however, VDAC1 overexpression did not change viability neither in +DOXY nor in −DOXY (Figure 5B). Moreover, the analysis of oxygen consumption revealed that VDAC1 increase did not ameliorate the respiratory profile of +DOXY cells. As shown in Figure 5C, ROUTINE respiration assayed in not permeabilized cells was not affected by the VDAC1 overexpression. Similar findings were observed in permeabilized cells, upon stimulation of OXPHOS and maximal ET capacity. Overall, these data strongly suggest that in +DOXY cells VDAC1 increment per se is not able to counteract mitochondrial bioenergetic impairment promoted by SOD1 G93A.

### 3.6. NHK1 Peptide Interacts with VDAC1

**Figure 6:**
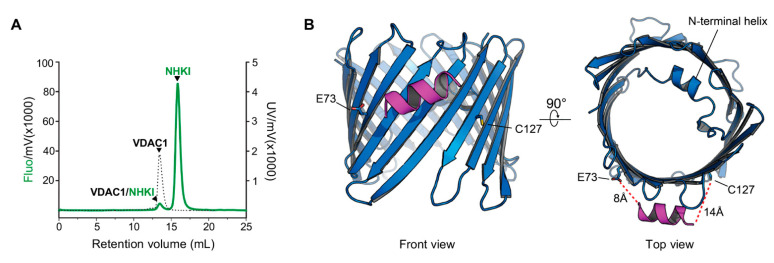
NHK1 peptide interacts with VDAC1 in a two-component system. (**A**) Analysis of VDAC1/NHK1-FITC interaction by FSEC. After incubation, complexes were separated by size-exclusion chromatography and detected by the fluorimeter. In green, the elution profile obtained at the emission wavelength of the FITC molecule; in dashed black, the elution profile obtained at 280 nm corresponding to the VDAC1 peak. (**B**) Molecular docking simulation result of the interaction between VDAC1 (in blue) and NHK1 peptide (in purple).

Despite several evidences, a direct interaction between NHK1 and VDAC1 has not been demonstrated yet. To this end, the recombinant VDAC1 was immobilized onto Ni^2+^-Sepharose beads and incubated with NHK1-FITC for 24 h, allowing the formation of complexes. After several washes, aimed at eliminating the unbound peptide, the development of such complexes was studied by fluorescence-detection size-exclusion chromatography (FSEC). As reported in Figure 6A, the fluorometer detected two peaks, one of which overlaps with the VDAC1 peak observed at 280 nm. This is the first biochemical evidence that NHK1 is able to bind VDAC1 in vitro in a two-components system. Moreover, this result suggests that the VDAC1-NHK1 complexes are somehow resistant to size-exclusion chromatography.

To identify a possible binding interface between VDAC1 and NHK1, a docking simulation was performed, using a generated model of NHK1 (NHK1_mod_) and the crystal structure of human VDAC1 (PDB:6G6U). The docking software produced 5000 poses of which 96 had a calculated ΔG value < −70.0. About 44% of those high-affinity poses located the NHK1_mod_ between VDAC β-strands 4 and 8, in the proximity of E73 and at only 14Å away from C127, the only cysteine residue exposed outside the barrel (Figure 6B). We have recently determined that in NSC34-SOD1 G93A cells, the C127 of VDAC1 is mostly found in a sulphonic acid over-oxidized form while a small proportion is present in a reduced form [60]. The unusual reduced form of C127 is thought to be a consequence of the destabilization of the VDAC1 structure due to deamidation of specific Asn and Gln residues found only in NSC34-SOD1 G93A cells [60].

## 4. Discussion

The aggregation of misfolded SOD1 mutants on the cytosolic surface of mitochondria is a distinctive feature of ALS affected MNs and strictly correlates with cell death and organelle dysfunction. In particular, SOD1 mutants impair several essential functions, such as the protein import [61], the physiological activity of resident proteins (i.e., those from the Bcl-2 family members and porins [20,62]) and the functioning of the respiratory chain complexes [12,16,38].

In the ALS-affected tissues, the specific interaction between SOD1 G93A and VDAC1 affects the metabolic trafficking across the OMM, as demonstrated in electrophysiological experiments with the recombinant proteins and confirmed in vivo [20,35,36,57]. Among the three mammalian isoforms, VDAC1 is the most conserved and abundant one [63]. Besides its involvement in the regulation of apoptosis, which has made this protein a widely studied pharmacological target in many diseases [64,65,66], the primary role of VDAC1 is to regulate mitochondrial bioenergetics. VDAC1 allows small hydrophilic metabolites and ions to be exchanged between mitochondria and the rest of the cell [21,29,30]. It has been recently estimated in the yeast that VDAC1 is the most representative protein of the OMM where it accounts for ~90% of the overall permeability [67,68]. The inactivation of the VDAC1 gene has drastic consequences for mitochondrial functionality: transcription of specific respiratory chain subunits encoded by mitochondrial DNA is abolished and oxidative phosphorylation is significantly reduced [68]. These events push the cell towards a metabolic re-arrangement aimed to by-pass mitochondrial involvement to produce energy [68]. VDAC1 also participates in the Ca^2+^ release in mitochondria from the ER, being part of the protein complexes involved in the formation of the contact sites between the organelles (the so-called mitochondrial-associated membranes, MAMs) [69,70]. As recently reported, the accumulation of mitochondrial SOD1 mutants inhibits the association of ER membranes with the mitochondria, affecting Ca^2+^ homeostasis [71]. In this perspective, the detachment of SOD1 mutant from mitochondria appears like a convincing strategy to recover the organelle bioenergetics and ameliorate the overall condition of MNs.

The small mitochondrial-targeted NHK1 peptide was designed to this purpose, using as a template the N-terminal domain of HK1, the most important physiological ligand of VDAC1. Previous experiments demonstrated that NHK1 prevents SOD1 G93A binding to the porin in a cell-free assay while, when expressed in a motor neuron-like cells, the peptide partially localizes at the mitochondria [36]. Considering these previous findings, we decided to treat with a synthetic NHK1 NSC34 cells stably expressing SOD1 G93A. This cellular model, indeed, shows a moderate aggregation of SOD1 mutant in mitochondria [18], as well as the typical bioenergetic impairments previously noticed in both ALS transgenic mouse model and other cell lines [38,57]. Furthermore, the hydrophobicity of the peptide due to the specific amino acid composition and the permeability experiments performed with a Transwell system made conceivable that NHK1 is able to cross through biological membrane.

The addition of increasing concentration of the peptide to +DOXY cells resulted in a dose-dependent reduction of toxicity mediated by SOD1 G93A. In addition, the treatment with the optimal dose of 10 μg/mL promoted a general improvement of the compromised mitochondrial respiration. The recovery of respiratory profile, however, was not linked to the rescue of complex I or II activity but rather to the specific increase of oxygen flows devoted to ATP synthesis and the reduction in proton leaking observed by monitoring the LEAK respiration. Overall, these data are consistent with an increase of ADP availability within mitochondria and, thus, with a significant increment of VDAC1 functionality.

VDAC1 is the main gateway for ADP/ATP exchanges through OMM and the preferential mitochondrial binding site for SOD1 mutant [20,72]. Our data clearly show that in +DOXY cells, treatment with NHK1 significantly reduces SOD1 G93A aggregates on the OMM, without disturbing the level and the subcellular distribution of the endogenous HK1, and promoting the overexpression of VDAC1. The resulting reduction of SOD1 G93A/VDAC1 toxic complexes at the organelle level enhances metabolic fluxes and, as previously demonstrated, recovers the mitochondrial membrane potential [36], the latter strictly dependent on metabolic exchanges through the porin. Since the dissipation of the mitochondrial membrane potential correlates with an increase of proton leak [73], our data about LEAK respiration confirm that NHK1 peptide increases VDAC1 functionality and, particularly, the adenylates exchanges.

However, if on one hand the mitochondrial SOD1 mutant reduction was predictable, as a confirmation of the interfering ability of NHK1 previously reported [36], the increase in VDAC1 levels was mostly unexpected. Nonetheless, HKs, and particularly the N-terminus, are known to modulate VDAC1 activity [37].

HK2 regulates the trafficking of newly synthesized VDAC1 towards mitochondria, as demonstrated by Dubey and colleagues. The same effect is not present when HK2 is deprived of its N-terminal domain [74]. Furthermore, the use of chemical inhibitors of HK2 (i.e., metformin) may vary the expression level of HK1 and VDAC1 in specific cell lines and/or conditions [75]. This suggests that HKs regulate VDAC1 trafficking and expression, at least under specific circumstances. Not coincidentally, variations in VDAC1 levels were observed exclusively in +DOXY cells, i.e., in the presence of SOD1 G93A, while the administration of NHK1 to −DOXY cells did not vary VDAC1 expression.

The susceptibility of +DOXY cells can be attributed to the overexpression of SOD1. It is known that SOD1 participates in the gene regulation during stress [76], and a direct link between its overexpression and the activation of VDAC genes was already observed in yeast [77]. In addition, growing evidence shows an active role of microRNAs for VDAC1 in neurodegeneration [78,79]. More recently, the dysregulation of specific miRNAs were correlated with the pathogenesis of ALS linked to SOD1 mutations [80]. Precisely, SOD1 G93A, G86S, and G17S mutants produce in NSC34 cells, in transgenic mouse or in ALS patients the miR-18b-5p downregulation and consequently HIF-1α upregulation. We have recently shown that HIF-1α is directly involved in the activation of the VDAC1 core promoter, both under basal conditions and cellular stress, and this leads to an increase in the corresponding transcripts [81]. Therefore, it is possible to speculate that the NHK1 peptide, in the presence of mitochondrial stress produced by the SOD1 mutant, triggers a cellular response through the activation of HIF-1α, possibly leading to the hyperactivation of the VDAC gene and, thus, to the overexpression of the protein. Also, the impaired activity of complex I induced by SOD1 G93A usually correlates with an increased oxidative stress (a well-known hallmark of ALS). In these conditions, an antioxidant response is induced by activating the expression of detoxifying-gene mediated by NRF2 [82], whose binding sites were recently identified on VDAC1 promoter [83].

In any case, the sole increase of VDAC1 has proven insufficient in recovery cell viability and the oxygen consumption as well, as highlighted by transfection experiments. These findings support the interfering properties of NHKI as responsible for its beneficial effects. In fact, only in the presence of the peptide, the increase of VDAC1 correlates with a significative rescue of SOD1 G93A toxicity. It is worth noting that VDAC1 is the main mitochondrial binding site of SOD1 mutants. Despite VDAC1 level is doubled in +DOXY cells treated with NHKI, there is not a concomitant increase in SOD1 G93A in the mitochondrial fraction. On the contrary, in this specific condition SOD1 mutant is significantly displaced from mitochondria. This reinforces the idea that the interaction between NHK1 and VDAC1 avoids the detrimental effect linked to SOD1 mutant. This concept is also strengthened by FSEC experiments as further proof of the direct interaction between the peptide and the porin.

Certainly, NSC34 cells represents a suitable model for HRR analyses due to SOD1 mutant mitochondrial accumulation and respiration impairment. At the same time, the relatively short duration of SOD1 G93A expression after the doxycycline addition does not allow in-depth studies relative to ROS accumulation or changes in mitophagy and/or mitochondrial biogenesis pathways. In this perspective, other experiments in primary cultures or transgenic mice expressing SOD1 G93A could be clarify any eventual additional role of NHK1 peptide. Another interesting aspect that deserves further investigations is establishing whether SOD1 G93A affects MAMs functionality through its direct interaction with VDAC1. In light of this hypothesis, we cannot exclude that the positive role exerted by NHK1, here observed, is partly due to the restoring of MAMs functionality.

Nonetheless, the beneficial effect of the NHK1 peptide on the NSC34-SOD1G93A cells is clear. The use of NHK1 and other interfering peptides could be a convincing strategy aimed at recovering mitochondrial dysfunction in ALS. Also, given that the interaction of misfolded proteins, such as α-synuclein and Aβ peptide, with VDAC1 represents a common mechanism shared by many other neurodegenerative disorders [84,85,86,87], it is plausible to expect that NHK1 might have similar beneficial effect in other diseases.

## Supplementary Materials

The following are available online at https://www.mdpi.com/article/10.3390/biomedicines9080948/s1, Figure S1: Characterization of NSC34 cell lines stably expressing SOD1 proteins. Figure S2: Activation of caspase-3 in NSC34-SOD1G93A. Figure S3: Respirometric protocol used in this work. Figure S4. Cellular uptake of MitoTraker Green. Figure S5: Analysis of VDAC1 expression. Table S1: Respiratory fluxes raw data.

## Abbreviations

ALS	amyotrophic lateral sclerosis
DOXY	doxycycline
ET	electron transport
FCR(s)	flux control ratio(s)
FITC	fluoresceine isothiocyanate
FSEC	fluorescence-detection size-exclusion chromatography
HK	hexokinase
HRR	high-resolution respirometry
MAMs	mitochondrial-associated membranes
MN(s)	motor neuron(s)
OMM	outer mitochondrial membrane
SOD1	Cu/Zn superoxide dismutase
VDAC	voltage-dependent anion channel
