# Supplementary material for: Small Hexokinase 1 Peptide against Toxic SOD1 G93A Mitochondrial Accumulation in ALS Rescues the ATP-Related Respiration
Source: Biomedicines. 2021 Aug 3;9(8):948. doi: 10.3390/biomedicines9080948 (PMC8392704; doi:10.3390/biomedicines9080948)
Supplement: Supplementary file 1 [file biomedicines-09-00948-s001.zip › biomedicines-1305095-supplementary.pdf]

biomedicines

Supplementary materials to:

Small hexokinase 1 peptide against toxic SOD1 G93A mitochondrial accumulation in ALS rescues the ATP-related respiration

Andrea Magrì [1,2†], Pierpaolo Risiglione [3†], Antonella Caccamo [4], Beatrice Formicola [5], Marianna Flora Tomasello [6], Cristina Arrigoni [7], Stefania Zimbone[1], Francesca Guarino [2,3], Francesca Re [5] and Angela Messina[1,2*]

[1] Department of Biological, Geological and Environmental Sciences, University of Catania, Via S. Sofia 64, 95123 Catania, Italy; andrea.magri@unict.it (A. Magrì); stefania_zimbone@libero.it (S.Z.); mess@unict.it (A. Messina)
[2] we.MitoBiotech S.R.L., C.so Italia 172, 95125 Catania, Italy
[3] Department of Biomedical and Biotechnological Sciences, University of Catania, Via S. Sofia 64, 95123 Catania, Italy; pierpaolo.risiglione@phd.unict.it (P.R.); fguarin@unict.it (F.G.)
[4] Department of Drug and Health Sciences, University of Catania, Via S. Sofia 64, 95123 Catania, Italy; acaccamo@gmail.com (A.C.)
[5] School of Medicine & Surgery, BioNanoMedicine Center NANOMIB, University of Milano-Bicocca, Via Cadore 48, Monza, 20900, Italy; b.formicola@campus.unimib.it (B.F.); francesca.re1@unimib.it (F.R.)
[6] Istituto di Cristallografia, CNR, Via Paolo Gaifami 18, 95126, Catania, Italy; mariannaflora.tomasello@cnr.it (M.F.T.)
[7] Department of Biology and Biotechnology, University of Pavia, Via Ferrata 9, 27100, Pavia, Italy; cristina.arrigoni01@unipv.it (C.A.)
* Correspondence: mess@unict.it
† These authors have equally contributed

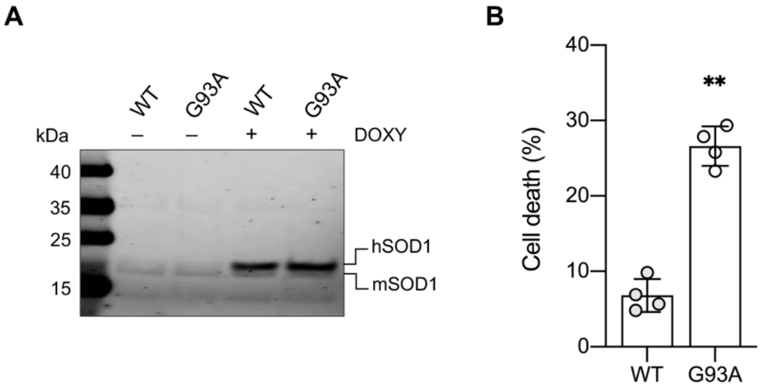

Figure S1. Characterization of NSC34 cell lines stably expressing SOD1 proteins. (A) Representative western blot image of total lysate of NSC34-SOD1WT and NSC34-SOD1G93A before and after doxycycline induction. Besides the presence of a lower band detected by the anti SOD1 antibody in all the sample and correspondent to the endogenous mouse SOD1 (mSOD1), a clear additional band was observed exclusively in +DOXY samples. The band correspond to the human myc-tagged SOD1 WT or G93A (hSOD1). A slight hSOD1 band could be detected also in -DOXY due to a basal expression. (B) Cell viability assay of +DOXY NSC34 cells expressing SOD1 WT or G93A. Data are normalized for the correspondent –DOXY cells and are expressed as means ± SD of n=5 independent experiments and analyzed by t-test with $^{**}p<0.01$ relative to WT sample.

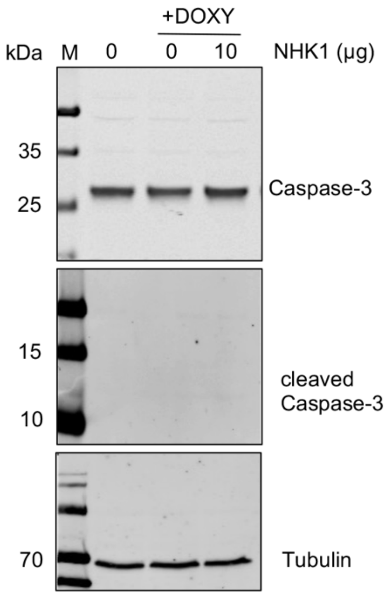

Figure S2. Activation of caspase-3 in NSC34-SOD1G93A. Representative western blot of total lysates from +/-DOXY NSC34-SOD1G93A untreated or treated with the NHK1 peptide. The total and cleaved caspase-3 were assayed as a marker of apoptosis. Tubulin was used as loading control.

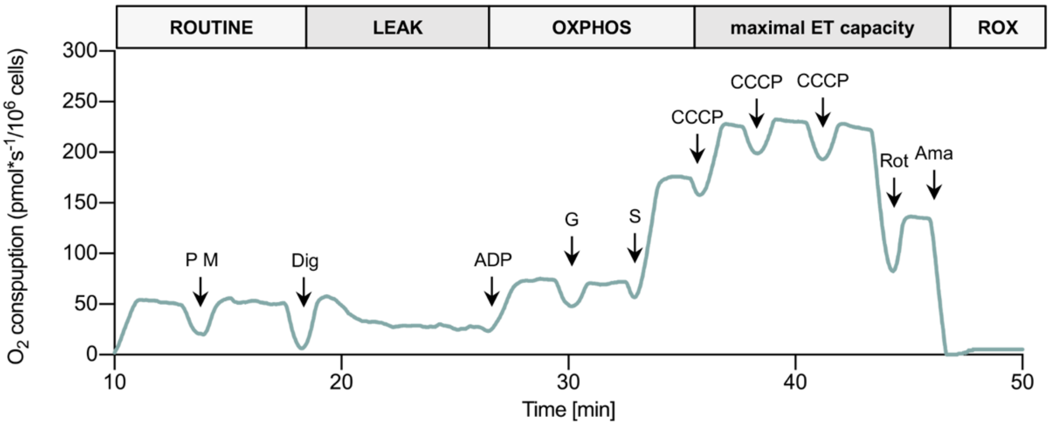

Figure S3. Respirometric protocol used in this work. Representative curve displaying the respirometric profile of -DOXY NSC34-SOD1G93A cells and the SUIT protocol applied. The respiratory states ROUTINE, LEAK, OXPHOS, ET, and ROX were achieved with the specific addition of substrates and inhibitors, as following: P, pyruvate; M, malate; Dig, digitonin; G, glutamate; S, succinate; CCCP, carbonyl cyanide 3-chlorophenylhydrazone; Rot, rotenone; Ama, antimycin.

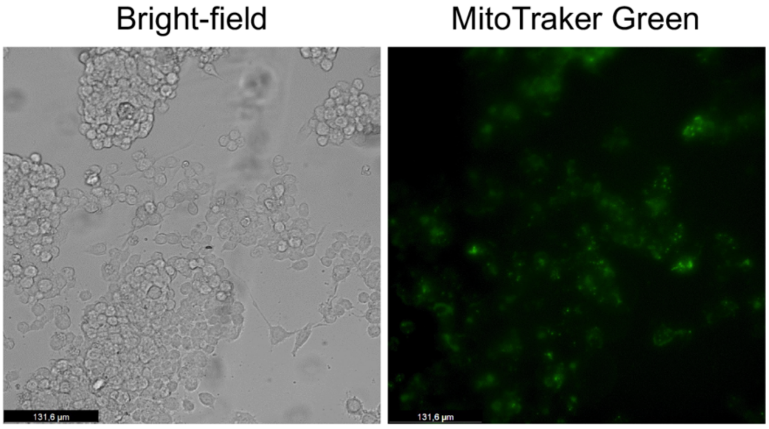

Figure S4. Cellular uptake of MitoTraker Green. Representative fluorescence microscopy image of NSC34-SOD1G93A (-DOXY) cells treated with MitoTraker Green.

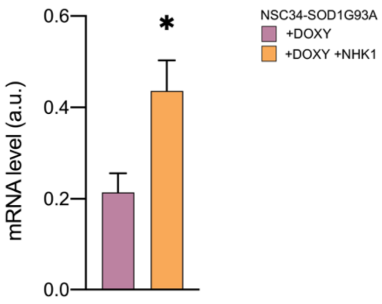

Figure S5. Quantitative PCR performed by Real-Time PCR on VDAC1 mRNA extracted from +DOXY NSC34-SOD1G93A cells treated with NHK1 peptide or DMSO. The housekeeping β-actin was used for normalization. Data are expressed as a mean ± SD of n=3 independent experiments and analyzed by t-test. Value of *$p<0.05$ was taken as significant.

	-DOXY	+DOXY	+DOXY +NHKI
ROUTINE	52.23 ± 1.8	46.09 ± 3.2	52.69 ± 3.4
LEAK	21.07 ± 2.7	21.58 ± 1.3	20.60 ± 2.8
OXPHOS (complex I)	75.81 ± 4.9	46.94 ± 6.7	49.93 ± 8.2
OXPHOS (complex I and II)	174.40 ± 14.4	125.62 ± 10.2	151.08 ± 8.4
ETS	208.22 ± 25.0	164.16 ± 16.4	191.09 ± 23.8
ETS (complex II)	116.73 ± 18.5	111.46 ± 9.6	123.88 ± 18.5

Table S1. Respiratory fluxes raw data. The ROX-corrected values of oxygen consumption relative to the respiratory states ROUTINE, LEAK, OXPHOS sustained by complex I, OXPHOS sustained by complex I and II (total OXPHOS), ETS and ETS sustained by complex II were obtained from -/+DOXY NSC34-SOD1G93A untreated or treated with NHK1 peptide. Data are expressed as pmol/second per million cells, as means ± SD of n=5 independent experiments
